# The physiological role of Homer2a and its novel short isoform, Homer2e, in NMDA receptor-mediated apoptosis in cerebellar granule cells

**DOI:** 10.1186/s13041-021-00804-8

**Published:** 2021-06-12

**Authors:** Teiichi Furuichi, Yuko Muto, Tetsushi Sadakata, Yumi Sato, Kanehiro Hayashi, Yoko Shiraishi-Yamaguchi, Yo Shinoda

**Affiliations:** 1 Laboratory for Molecular Neurogenesis, RIKEN Brain Science Institute, Wako, Saitama 351-0198 Japan; 2 JST-CREST, Kawaguchi, Saitama 332-0012 Japan; 3 Present Address: Department of Applied Biological Science, Faculty of Science and Technology, Tokyo University of Science, Noda, Chiba 278-8510 Japan; 4 Present Address: Education and Research Support Center, Gunma University Graduate School of Medicine, Maebashi, Gunma 371-8511 Japan; 5 Present Address: Laboratory of Proteome Research, Laboratory of Proteomics for Drug Discovery, Center for Drug Design Research, National Institutes of Biomedical Innovation, Health and Nutrition, Ibaraki, Osaka 567-0085 Japan; 6 Present Address: Department of Anatomy, Keio University School of Medicine, Shinjuku-ku, Tokyo, 160-8582 Japan; 7 Present Address: Department of Developing Human Resources for R&D Programs, Japan Science and Technology Agency (JST), Chiyoda-ku, Tokyo, 102-8666 Japan; 8 Present Address: Department of Environmental Health, School of Pharmacy, Tokyo University of Pharmacy and Life Sciences, Hachioji, Tokyo, 192-0392 Japan

**Keywords:** Homer2, Cerebellar granule cell, NMDAR, Excitotoxicity

## Abstract

Homer is a postsynaptic scaffold protein, which has long and short isoforms. The long form of Homer consists of an N-terminal target-binding domain and a C-terminal multimerization domain, linking multiple proteins within a complex. The short form of Homer only has the N-terminal domain and likely acts as a dominant negative regulator. Homer2a, one of the long form isoforms of the Homer family, expresses with a transient peak in the early postnatal stage of mouse cerebellar granule cells (CGCs); however, the functions of Homer2a in CGCs are not fully understood yet. In this study, we investigated the physiological roles of Homer2a in CGCs using recombinant adenovirus vectors. Overexpression of the Homer2a N-terminal domain construct, which was made structurally reminiscent with Homer1a, altered NMDAR1 localization, decreased NMDA currents, and promoted the survival of CGCs. These results suggest that the Homer2a N-terminal domain acts as a dominant negative protein to attenuate NMDAR-mediated excitotoxicity. Moreover, we identified a novel short form N-terminal domain-containing Homer2, named Homer2e, which was induced by apoptotic stimulation such as ischemic brain injury. Our study suggests that the long and short forms of Homer2 are involved in apoptosis of CGCs.

## Introduction

Homer proteins are scaffold proteins that predominantly exist in the postsynaptic density (PSD) of neurons and tether a variety of target proteins within the PSDs, including the group I metabotropic glutamate receptor 1α/5 (mGluR1α/5), inositol 1,4,5-trisphosphate receptor (IP_3_R), and Shank, a scaffold protein for the N-methyl-D-aspartate receptor (NMDAR) complex [1–4]. There are three distinct genes in the Homer family, Homer1/vesl-1, Homer2/Cupidin/vesl-2, and Homer3, and they are classified into two major forms, long and short [5, 6]. Long Homers, such as Homer1b/c/d, 2a/b, and 3a/b, consist of two main domains, the N-terminal enabled/vasodilator-stimulated phosphoprotein homology 1 (EVH-1) domain for the target binding and the C-terminal coiled-coil domain for the self-multimerization [7]. However, the short Homer, such as Homer1a, which has been reported as an immediate-early gene induced by electro-convulsive seizure in an activity-dependent manner [8], only has the N-terminal target-binding domain. Thus, Homer1a is thought to act as a natural dominant negative regulator by competing with long Homer proteins that form Homer-target protein multimers via the C-terminal domain [9, 10]. In addition, Homer1a is known to promote cell protection from apoptosis [11–13].

We have reported that Homer2a (also known as Cupidin), a long Homer isoform, has an expression pattern that peaks around postnatal day 7 during mouse cerebellar development [14] and is highly expressed in cerebellar granule cells (CGCs) [15]. In the postnatal stage with high Homer2a expression, a significant apoptotic event of CGCs is observed [16]. Additionally, the development and survival of CGCs is associated with glutamatergic activity via NMDAR [17]. It has also been reported that glutamate stimulation leads to the postsynaptic targeting of Homer2a and its declustering in CGCs [18]. Therefore, Homer2a may be associated with developmental events of CGCs including apoptosis, however, its physiological role has not been fully elucidated.

In this study, we investigated the physiological role of Homer2a and found its novel short isoform, Homer2e. We analyzed the effects of Homer2a on the clustering of NMDAR, and on NMDA currents and NMDA-induced apoptosis in CGCs. We also described the ischemia-associated induction of the novel alternative splicing Homer2 isoform, Homer2e, in CGCs.

## Materials and methods

### Cerebellar primary culture and oxygen–glucose deprivation (OGD)

Primary dissociated CGCs were prepared from fetal ICR mice (Nihon SLC, Hamamatsu, Japan) as described previously [19]. Briefly, cerebella from 18-day-old embryos were trypsinized with 1% trypsin (Sigma-Aldrich, St. Louis, MO, USA) and 500 units/mL DNase I (Sigma-Aldrich) for 13 min at 37 °C and were further triturated by repeated passage through a fine-tipped pipette in Ca^2+^-free Hank’s balanced salt solution (HBSS; Sigma-Aldrich) containing 500 units/mL DNase I and 12 mM MgSO_4_. Dispersed cells were plated at a density of 35 × 10^4^ cells/cm^2^ on poly-l-lysine-coated glass cover slips (Matsunami, Osaka, Japan), and then cultured in serum-free Eagle’s medium supplemented with 1 mg/mL bovine serum albumin, 10 μg/mL bovine insulin, 0.1 nM l-thyroxine, 0.1 mg/mL human transferrin, 1 μg/mL aprotinin (all from Sigma-Aldrich), 30 nM sodium selenite (Merck, Darmstardt, Germany), 0.25% glucose (Nacalai Tesque, Kyoto, Japan), 2 mM glutamine (Nacalai Tesque), 100 μg/mL streptomycin (Meiji Seika, Tokyo, Japan), and 100 U/mL penicillin (Banyu Pharmaceutical, Tokyo, Japan). The cultures were maintained at 37 °C in a humidified incubator with 5% CO_2_, and half the volume of the culture medium was replaced with fresh medium once a week. The cultures were used at 12–15 days in vitro (DIV). Oxygen–glucose deprivation experiment was carried out using AnaeroPack (MGC, Tokyo, Japan) according to the manufactures’ instructions with Eagle’s medium in the absence of glucose and incubated the cells for 90 min at 37 °C in a humidified incubator with 5% CO_2_.

### Generation and infection of adenoviruses expressing EGFP-Homer2a constructs

The enhanced green fluorescent protein (EGFP)-coding region of pEGFP-C1 (Clontech, Cambridge, UK) was fused in frame to the Homer2a N-terminus or to mutants to generate EGFP-fused Homer2a constructs. Replication-deficient adenoviruses were generated with the COS-TPC method as described previously [20]. Briefly, the EGFP-tagged full-length (H2a-F), C-terminal (H2a-C), and N-terminal domain of Homer2a (H2a-N) were cloned into the SwaI site of the pAxCAwt cosmid cassette (Takara, Tokyo, Japan). The recombinant adenoviruses (AdVs) were generated by homologous recombination between an *Eco*T22I-digested Ad5-dlx DNA-terminal protein complex and recombinant cosmid vectors in HEK293 cells. The generated AdVs were propagated in HEK293 cells. The AdVs were concentrated and purified by double cesium chloride gradient centrifugation and the titers of AdVs were measured by the 50% tissue culture infectious dose method [21]. Cultured CGCs at 12 DIV were exposed to AdVs at a multiplicity of infection = 30. Infected CGCs were analyzed 48 h after the infection (14 DIV).

### Immunocytochemistry

Immunocytochemistry was performed as described previously with minor modifications [22]. Briefly, cells were fixed with 4% paraformaldehyde in 0.1 M phosphate buffer, permeabilized in methanol for 30 min at − 30 °C, preincubated with 5% normal donkey serum in PBS for 1 h, and then incubated with the primary antibodies, rabbit anti-Homer2a antibody [14] or mouse anti-NMDA receptor 1 (NR1) antibody (BD Transduction Laboratories, Lexington, KY, USA), for 16 h at 4 °C. After washing with PBS, the samples were incubated with Alexa-conjugated secondary antibodies (Thermo Fisher Scientific, Waltham, MA, USA). Coverslips were mounted in Vectashield (Vector Laboratories, Burlingame, CA, USA) mounting medium. Immunofluorescence was observed using a Meta-510 confocal laser microscope (Zeiss, Oberkochen, Germany).

### Western blotting

Western blotting was performed as described previously with minor modifications [23]. Total cell lysates prepared from CGC cultures were boiled in SDS-PAGE sample buffer. Samples were separated by SDS-PAGE gels and blotted onto a polyvinylidene difluoride membranes. Proteins on the membranes were immunodetected with rabbit anti-Homer2a, rabbit anti-pan Homer [15]. The immunoblots were incubated with horseradish peroxidase-conjugated secondary antibodies (KPL, Gaithersburg, MD, USA) and developed with ECL reagents (Amersham, Buckinghamshire, UK). The chemiluminescence was imaged by a LAS-3000 imaging system (Fujifilm, Tokyo, Japan). The band intensity was quantified with Image J (National Institutes of Health).

### Electrophysiology

Whole-cell patch clamp recordings were obtained using a partly modified previously reported method [24]. AdV-infected CGCs on glass coverslips at 14 DIV (2 days after infection at 12 DIV) were transferred into the experimental chamber and superfused with modified Krebs–Ringer solution (mM): 145 NaCl, 5 KCl, 2 CaCl_2_, 10 glucose, 10 HEPES, 0.01 glycine (pH 7.4 with NaOH). Tetrodotoxin (1 μM), bicuculline (10 μM), and NBQX (10 µM) were added to block action potential, GABAergic transmission, and non-NMDAR-mediated glutamatergic transmission, respectively. NMDA (50 µM) and AP5 (50 µM) were applied to elicit and block the NMDA current, respectively. All agonists and antagonists were purchased from Tocris (Tokyo, Japan). The experimental chamber, consisting of an acrylic frame with a glass bottom, was mounted on the stage of an inverted Eclipse TE2000-U phase contrast microscope (Nikon, Tokyo, Japan). Patch pipettes were made from glass capillaries (Clark Electromedical Instruments, Pangbourne, UK) using a P-97 horizontal puller (Sutter Instrument, Novato, CA, USA). The pipette had a direct current resistance of 4–7 Ω when it was filled with the following solution (mM): 140 D-glucuronate, 7 CsCl, 155 CsOH, 5 EGTA, and 10 HEPES (pH 7.2 with CsOH). The pipette was connected to patch clamp amplifier AXOPATCH 200B (Molecular Devices, San Jose, CA, USA) and filtered with a 1 kHz Bessel low-pass filter.

### Cell survival assays

The MTT assay was performed using MTT Kit I (Boehringer Mannheim, Mannheim, Germany) according to the manufacturer’s instructions. Briefly, 10 μL of the 5 mg/mL MTT labeling reagent was added to 100 μL of neuronal cultures in each well of 96-well culture plates, and the plates were incubated for 4 h at 37 °C in a humidified incubator with 5% CO_2_. Then, 100 μL of the solubilizing solution was added to each well and further incubated overnight. Absorbance of the solubilized samples was measured at 570 nm and 700 nm (reference wavelength). The extent of MTT conversion in cells was expressed as a percentage of the control.

### Preparation of brain ischemia samples

Transient global cerebral ischemia was induced using the bilateral occlusion of the common carotid arteries technique as previously described [25]. ICR mice at P56 were anesthetized and an incision was made in the ventral neck to expose the common carotid arteries. Brain ischemia was induced by bilateral occlusion of the common carotid arteries using aneurysm clips for 20 min. At the end of each occlusion, the aneurism clips were removed, and the arteries were visually inspected for reperfusion. The incision was then closed with sutures.

### Nested PCR, molecular cloning, and RT-PCR of *Homer2* isoforms

The *Homer2* isoforms were cloned by the nested PCR method using a mouse brain cDNA library and the following primers: the outer primer set, 5′ primer, Homer2a-5′-exon2-fw (5′-TCACCAGGAACAGCTATCGG-3′, corresponding to exon2 of *Homer2a*), and 3′ primer, Homer2a-3′-UTR-rev (5′-TCTGGAGACAGACAGATCGC-3′, corresponding to the 3′ untranslated region of *Homer2a*), and the inner primer set, 5′ primer, Homer2a-5′-exon3-fw (5′-CGGTTTGGGATTCTCCTCCG-3′, corresponding to exon3 of *Homer2a*), and 3′ primer, Homer2a-3′-exon9-rev (5′-TTTGATTGTCTCTTTCGGCC-3′, corresponding to exon9 of *Homer2a*). Amplified DNA fragments were cloned into the pCR4-TOPO vector (Thermo Fisher Scientific) and sequenced. For RT-PCR, the TRIzol Reagent (Thermo Fisher Scientific) was used to prepare total RNA from cerebella of either normal or ischemia-induced mice (ICR at P56) and from CGC cultures that were untreated or subjected to oxygen and glucose deprivation for 60 min. RNA samples were digested with RQ1 RNase-free DNase (Promega, Madison, WI, USA) for 30 min at 37 °C. The resulting DNA-free RNAs were used to synthesize cDNAs using SuperScript II reverse transcriptase (Thermo Fisher Scientific) and oligo dT. PCR of the cDNAs was performed with an ExTaq polymerase kit (Takara) with specific primers bridging between exon4 and exon6 of *Homer2a*. The PCR products were separated on 2% agarose gel and visualized using a UV transilluminator (Bio-Rad, Tokyo, Japan) after staining with ethidium bromide.

### Statistical analysis

Data are presented as the mean ± SEM. Comparisons of multiple data were statistically evaluated by one-way analysis of variance (ANOVA) and post hoc Bonferroni test.

## Results

### Overexpression of exogenous Homer2a in CGCs

**Fig. 1:**
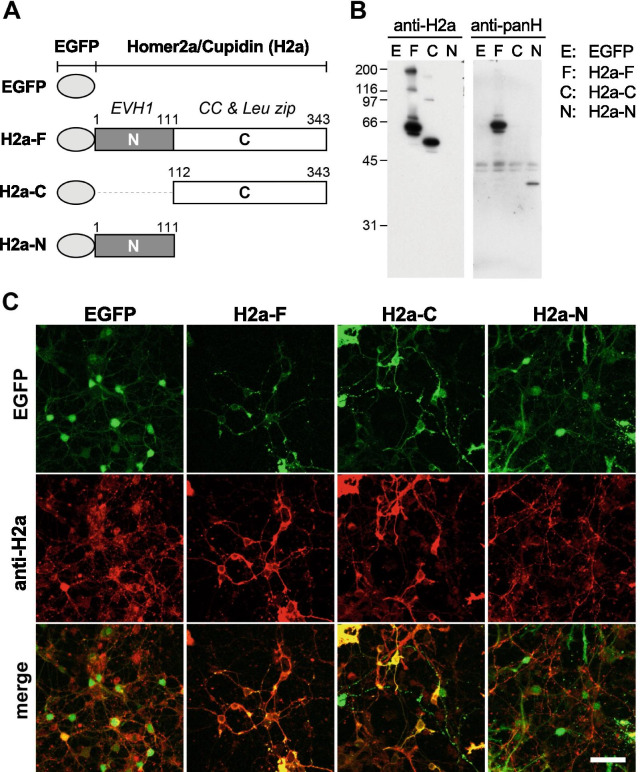
Overexpression of the Homer2a constructs in CGCs and their subcellular distributions. **A** Schematic image of the AdV-vector constructs of Homer2a. H2a-F, full-length Homer2a; H2a-C, carboxy-terminal side of Homer2a; H2a-N, amino-terminal side of Homer2a. **B** Immunoblotting of Homer2 in CGCs infected with AdV-vector constructs. Equal amounts of protein were loaded in each lane and immunoblotted with antibodies against Homer2a and pan-Homer. **C** Subcellular distribution of each Homer2a variant expressed in CGCs. Scale bar, 50 µm

To clarify the function of Homer2a in CGCs, four recombinant AdV vectors were constructed (Fig. 1A): AdV carrying EGFP only, and three AdV constructs consisting of EGFP fused to full length (H2a-F), C-terminal (containing residues 112–343; H2a-C) or N-terminal (containing residues 1–111; H2a-N) Homer2a, which structurally resemble a short form of Homer, like Homer1a.

We first confirmed that all the Homer2a proteins were overexpressed in CGCs by Western blotting (Fig. 1B). Cultured CGCs were infected with AdVs at 12 DIV and were analyzed 48 h after infection. The anti-Homer2a antibody, which binds to the C-terminus of Homer2a, immunodetected H2a-F and H2a-C but not the H2a-N, because it lacked the epitope for this antibody (Fig. 1B, left). The anti-panHomer antibody, which binds to the N-terminus of all Homer family isoforms, detected H2a-F and H2a-N but not H2a-C (Fig. 1B right). These results indicated that the recombinant AdV-Homer2a infection successfully induced the overexpression of Homer2a proteins in CGCs.

Next, we analyzed the subcellular localization of endogenous Homer2a and its overexpressed mutants in CGCs (Fig. 1C). The endogenous Homer2a was observed in cultured CGCs mostly in a punctate pattern at postsynaptic sites as we previously reported [14, 18]. In CGCs overexpressing EGFP-fused H2a-F, EGFP fluorescence were observed as punctate pattern along dendrites, which is thought to be postsynaptic sites. However, when EGFP-fused H2a-C and H2a-N were overexpressed, EGFP fluorescence were observed mainly in soma and neurites with punctate pattern along dendrites.

### Colocalization of Homer2a and NMDAR

**Fig. 2:**
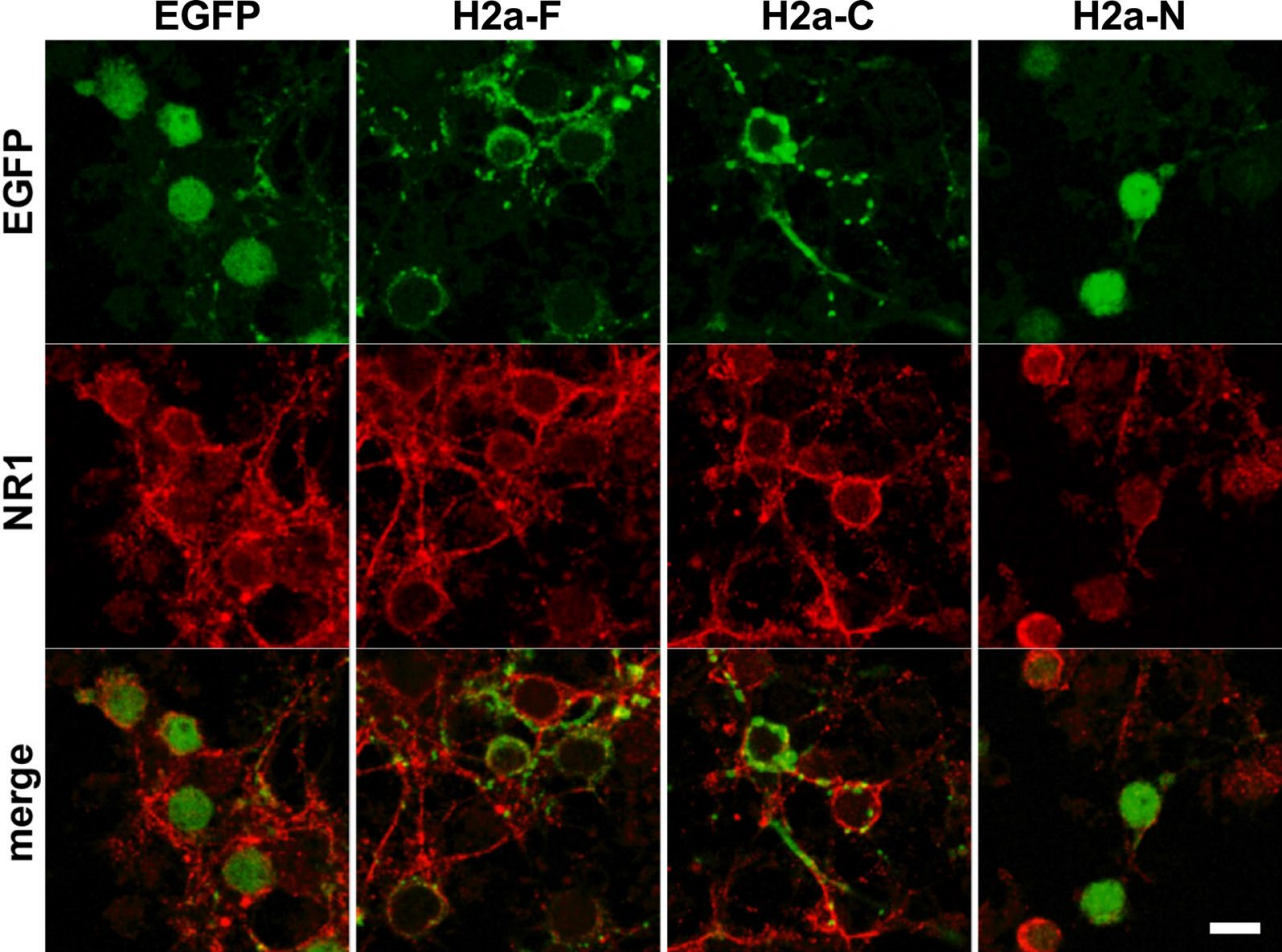
The colocalization of Homer2 mutants and NR1. EGFP and NR1-immunostained CGCs overexpressing Homer2 variants. Scale bar, 10 µm

Because Homer2a binds and regulates postsynaptic proteins [14, 26], we examined the colocalization of postsynaptic glutamate receptor NR1 and Homer mutants in CGCs (Fig. 2). The immunoreactivity for recombinants of Homer2 appeared to be localized at the area immunoreactive to NR1 with punctate pattern. On the other hand, the punctate immunoreactivity of NR1 was disappeared in H2a-N overexpressed CGCs. These data suggest that Homer2a overexpression alters the localization of NR1, which may affect the physiological phenotype of CGCs.

### NMDA currents decrease upon overexpression of the N-terminal domain of Homer2a

**Fig. 3:**
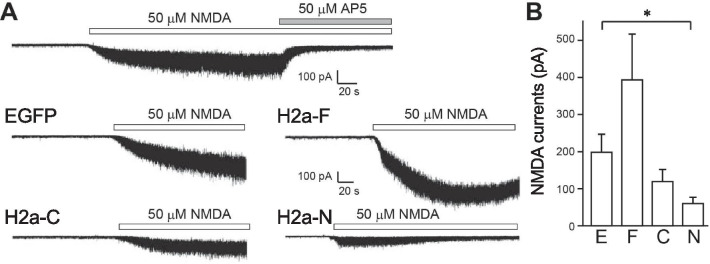
NMDAR currents were decreased by overexpression of the N-terminal domain of Homer2a. **A** Top row: representative NMDA-induced inward current trace from control (non-AdV-infected) CGCs. Middle and bottom rows: representative current traces from CGCs infected with EGFP or Homer2 constructs. Whole cell recordings were performed 48 h after infection. **B** Quantification of NMDA currents. Infection with H2a-N caused a significant decrease in the NMDA current compared with the EGFP group. E: EGFP, F: H2a-F, C: H2a-C, N: H2a-N. *p < 0.05, one-way ANOVA and post hoc Bonferroni test. n = 3

NMDAR is a calcium permeable glutamate receptor, which is related to several critical cellular events such as synaptic plasticity, cellular differentiation, and apoptosis [27]. To examine the effects of Homer2a overexpression on the physiological response of NMDAR, we carried out whole-cell patch-clamp recordings on CGCs (Fig. 3). In naive CGCs, application of 50 μM NMDA under voltage clamp at − 60 mV elicited inward currents that were blocked by AP5, an NMDAR antagonist (Fig. 3A). We also recorded these NMDA currents in AdV-infected CGCs (Fig. 3A, B). Compared with EGFP-expressing CGCs, in H2a-F-overexpressing CGCs the amplitude of NMDA currents tended to be increased. However, consistent with the altered localization of NR1, H2a-N-overexpressing CGCs showed a significantly lower NMDA current amplitude (Fig. 3B).

### Overexpression of the Homer2a N-terminal domain suppresses NMDA-mediated cell death of CGCs

**Fig. 4:**
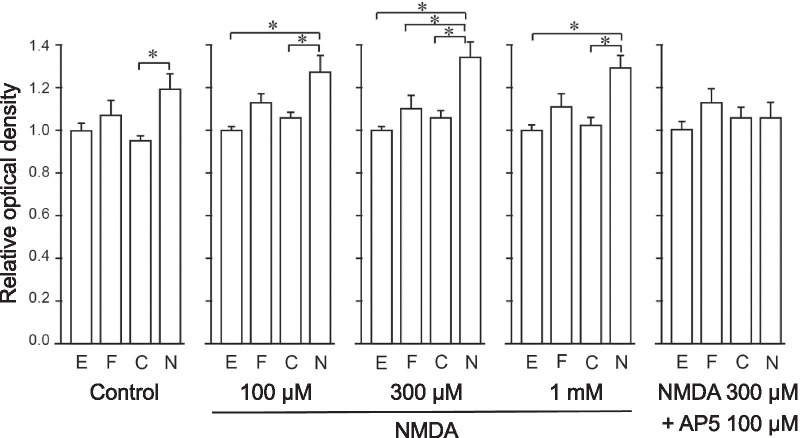
Overexpression of the Homer2a N-terminal domain suppressed NMDA-induced apoptosis. Normalized optical density of MTT assays of CGCs overexpressing EGFP or Homer2 constructs after the indicated NMDA treatment. NMDA-induced cell death was inhibited in H2a-N-overexpressing CGCs under all doses of NMDA. E: EGFP, F: H2a-F, C: H2a-C, N: H2a-N. *p < 0.05, one-way ANOVA and post hoc Bonferroni test; n = 3

Changes in NMDAR localization and activity are associated with cell apoptosis [17]. Therefore, we examined the cell viability of AdV-infected CGCs after NMDA administration. We treated the cells with different doses of NMDA with or without AP5 and analyzed the NMDA-induced cell death of H2a-F, -C or -N-overexpressing CGCs by the MTT assay (Fig. 4). Among these cells, the relative MTT values of H2a-N-overexpressing CGCs were the highest after treatment with all NMDA doses (100 μM, 300 μM, and 1 mM), however this difference was diminished by co-application of AP5. These results indicated that H2a-N-overexpressing CGCs were more resistant to NMDAR-mediated excitotoxicity than EGFP, H2a-F, and H2a-C-overexpressing CGCs.

### The newly identified short isoform, Homer2e, is induced by ischemia

**Fig. 5:**
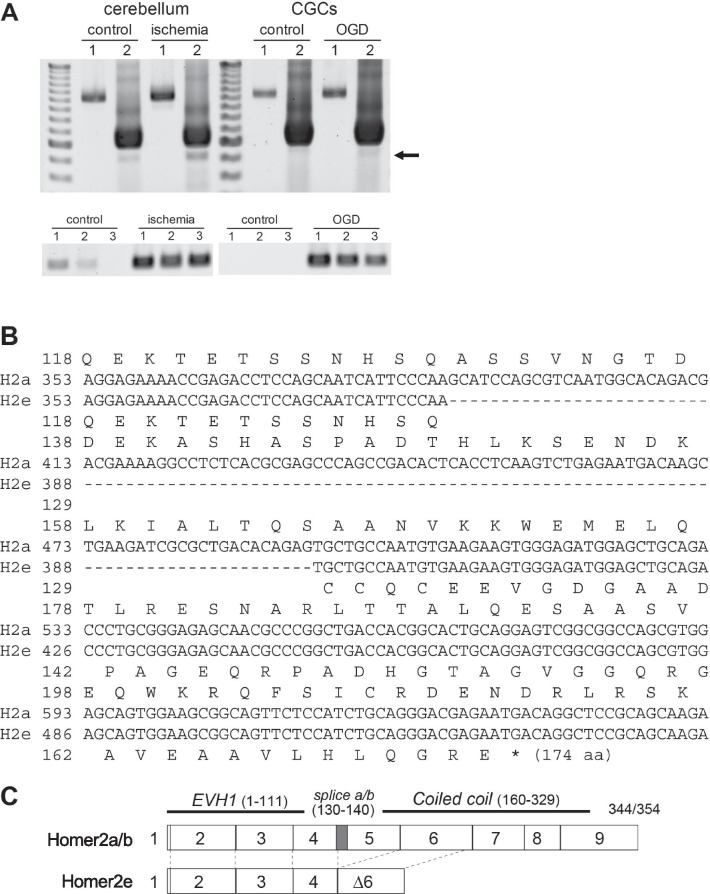
Identification of a new Homer2 variant, Homer2e, induced by ischemia in mouse cerebellum and by OGD in CGCs. **A** Upper, nested PCR of mouse cerebellum cDNA performed on the coding region of *Homer2a*. The first and the second PCR reactions are depicted as 1 and 2. In the second PCR, a small size band just underneath the major band was detected particularly in ischemic and OGD samples (arrow). Lower, RT-PCR detection of *Homer2e* in the cerebellum and CGCs. *Homer2e* increased in both ischemic cerebellum and OGD CGCs. Three individual animals (n = 3) and cultures (n = 3) were analyzed in each condition. **B** Comparison of the partial nucleotide and deduced amino acid sequences between Homer2a and Homer2e. **C** Comparison of exons between Homer2a/b and Homer2e. Exons 5 and 7–9 of Homer2a/b are lacking in Homer2e. Because the nucleotide deficits cause a frameshift, Homer2e encodes a potential protein of 174 amino acids, which lacks the C-terminal of Homer2a/b

Because overexpression of H2a-N, a Homer1a-like short Homer2a, exerted resistance to NMDAR-induced apoptosis of CGCs, it is possible that apoptosis-dependent expression of short Homer2 proteins can function as an apoptosis-resistance molecule, just like Homer1a [11–13]. To examine whether any of short Homer2 isoforms are expressed in apoptotic CGCs, we performed nested and RT-PCR analysis of *Homer2* mRNA expressed before and after apoptosis induction (Fig. 5). In the cerebellum of mouse ischemic brain, we detected by nested PCR a *Homer2*-derived band (Fig. 5A). The same nested PCR product was also obtained from cultured CGCs with OGD, which induces apoptosis in CGCs [28]. Sequence analysis identified this product as an alternative splicing variant of Homer2a containing a frame shift that occurred by skipping exon5 and a part of exon6 (Fig. 5B, C). The open reading frame predicted for this novel product, named Homer2e, is composed of 174 amino acids, including the entire N-terminal target-binding domain, but lacking the C-terminal domain of Homer2a. This frame-shifted C-terminus domain of Homer2e (CCQCEEVGDGAADPAGEQRPADHGTAGVGGQRGAVEAAVLHLQGRE) did not match any protein domains from conserved domain sequence database in NCBI. Thus, this novel isoform is thought to be structurally similar to the short Homer1a, and may act as a dominant negative of long Homer2a.

## Discussion

Our study showed that overexpression of the Homer2a N-terminal domain decreased NR1 localization and the NMDA currents, and had an antiapoptotic function in CGCs. Moreover, we identified a new short Homer2 isoform, Homer2e, which was upregulated in response to apoptotic conditions and ischemic brain injury.

**Fig. 6:**
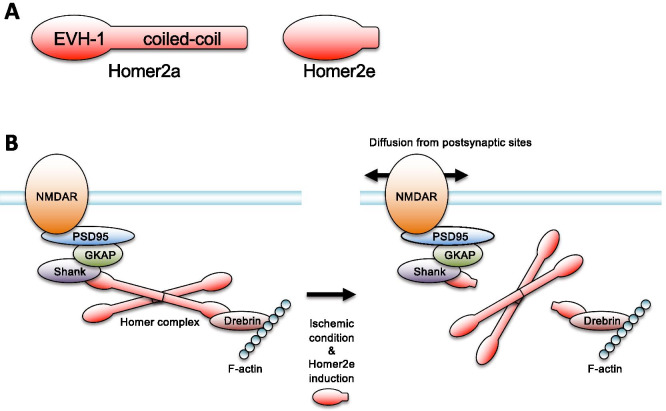
Schematic diagram of Homer2a/2e and their possible role. **A** Schematic structure of Homer2a and 2e. Long Homer2a has EVH-1 domain in N-terminal and coiled-coil domain in C-terminal. Homer2e has only EVH-1 domain and lack of coiled-coil domain. **B** Schematic diagram of the possible role of Homer2a and 2e. Left: Long Homer2a multimerizes using their C-terminus coiled-coil domain and binds to Shank and Drebrin using their N-terminus EVH-1 domain to stabilize NMDAR at the postsynaptic site via GKAP, PSD95 and F-actin complex. Right: Ischemic condition induces Homer2e and it prevents the binding between Homer2a and Shank/Drebrin by takeover their EVH-1 binding sites, which enhances declustering of the protein complex including NMDAR in the postsynaptic site

Because of the relationship between Homer proteins and cell apoptosis [29], the representative short form Homer, Homer1a, has been extensively studied as a dominant negative regulator for apoptosis. For example, Homer1a blocked tumor necrosis factor-a/cycloheximide-induced apoptotic cell death via the mitogen-activated protein kinase pathway in PC12 cells [13]. In retinal ganglion cells, retinal ischemia and reperfusion induced Homer1a expression, showing neuroprotective effects [12]. Knockdown of Homer1a reduced the cytoprotective effect of Chikusetsu saponin IVa, which controls the reactive oxygen species and intracellular Ca^2+^ homeostasis in cardiomyocytes [30]. Overexpression of Homer1a inhibited the PI3K/AKT/mTOR signaling pathway, enhanced autophagy and cell viability of rat cortical neurons [31]. Homer1a is upregulated in reactive astrocytes and protects astrocytes from apoptosis [11]. Additionally, upregulated Homer1a in astrocytes downregulated astrocytic glutamate release by precluding the mGluR5-mediated intracellular Ca^2+^ signaling [11]. In contrast, the long Homer1 isoform, Homer1b and c, have been reported as promoters of neuronal apoptosis via a Bax/Bcl-2-dependent pathway [32]. Furthermore, the other long Homer isoforms, Homer2a, has been shown to be involved in apoptosis of cultured endothelial cells [33].These studies indicate that Homer proteins are associated with several signaling pathways related to apoptosis in neuronal and non-neuronal cells, and especially short Homer proteins may play an important role in protection from apoptotic cell death. In the present study, we addressed the possible role of Homer2e, which is induced via ischemic condition, disassembles postsynaptic Homer-Shank-NMDAR clusters, attenuates excess calcium influx, and prevents apoptosis in CGCs (Fig. 6).

Long Homer proteins have two characteristic structural domains, the N-terminal EVH-1 domain and the Homer-specific C-terminal domain, whereas short Homer proteins lack the C-terminal domain [5]. The EVH-1 domain binds to the proline-rich amino acid sequence, Pro-Pro-X-X-Phe, which is found in many target proteins including the Shank protein family [34]. Shank proteins are scaffold postsynaptic proteins that directly or indirectly bind to several postsynaptic proteins including NMDAR, to tether them to specific postsynaptic sites [35]. The C-terminal domain of Homer consists of a coiled-coil structure followed by two leucine zipper motifs, which form homo- or heteromeric interactions between long Homers [1]. Because short Homers lacking the C-terminal domain cannot interact with other Homer proteins, short Homer proteins are thought to act as dominant negative regulators of long Homer proteins, thereby affecting the distribution of postsynaptic proteins to alter the physiological functions of postsynapses. In fact, overexpression of recombinant Homer1a decreased the clusters of PSD95, NR1, and GluR2, a subunit of AMPA type glutamate receptor, and reduced NMDAR and AMPAR-mediated postsynaptic currents [36]. Additionally, the activity-dependent expression of Homer1a induced rapid de-clustering of Homer1c as well as PSD95 and GluR2 [37]. Although there is difference between Homer1 and 2 in the cellular and regional expression pattern in the brain [15] and phenotypes of knockout mice [38], the short Homer2e may act as a natural dominant negative regulator of the long Homer2a/b. In the present study, we did not measure the protein expression level of Homer2e because lack of specific antibody. If Homer2e can act as a dominant negative of long Homer2, protein expression level of Homer2e should be considered. In addition, historical publications reported that short Homer1a protein level is very low compared to the constitutively expressed Homers, even when mRNA induction is quite robust [39]. Therefore, it is possible that the protein expression level of induced Homer2e is also low compared with other constitutively expressed Homer2 isoforms. One of the possible explanations of how low-level expressed protein can act effectively as a dominant negative is their specific localization. Since Homer proteins are existing in postsynaptic site, if induced short Homers are accumulated or locally translated at postsynaptic site, the local concentration of these short Homers can compete pre-existing long Homers. Therefore, one of the possible and attractive roles of the short Homer2e may be the candidate protein associated with synaptic tagging as Homer1a [40]. The basal and induced protein expression level of Homer1a is known to be very low compared to the constitutively expressed long Homers [39]; however, it can be act as a dominant negative in the specific postsynaptic site as synaptic tag.

In addition to the declustering of postsynaptic proteins targeted to N-terminus domain of long Homer2, the proteins targeted to C-terminus domain of long Homer2 are also to be considered as the regulated proteins by Homer2e. Cdc42 is known as a small GTPase of the Rho family, which modulates cell morphology, migration, endocytosis and cell cycle [41], which binds C-terminus domain of Homer2a [26]. Since EVH-1 domain on Homer2a can bind Ophn-1 (GTPase activating protein) and beta-PIX (guanine nucleotide exchange factor) [26], the disruption of the link between Ophn-1/beta-PIX and Cdc42 may affect actin polymerization and subsequent dendritic spine morphology. However, it remains to be elucidated whether short Homer2e plays a role as a dominant negative regulator of the long Homer2 isoform.

In conclusion, our study showed that Homer2a is involved in the regulation of NMDAR currents and plays a role in NMDAR-mediated cell physiology including apoptosis. The novel short isoform, Homer2e, is induced under apoptotic conditions and possibly acts as a dominant negative regulator of the long form of Homer2, i.e., decreases the NMDAR currents, probably resulting in the repression of NMDAR-elicited excitotoxicity and apoptosis. The harmonization between the long and short Homer isoforms in the developing cerebellum may be crucial for fine physiological regulation of CGCs.

## Data Availability

All data generated or analyzed during this study are included in this published article.

## Abbreviations

CGCs: Cerebellar granule cells
NMDAR: N-methyl-D-aspartate receptor
NR1: NMDA receptor subtype 1
PSD: Postsynaptic density
mGluR: Metabotropic glutamate receptor
IP_3_R: Inositol 1,4,5-trisphosphate receptor
EVH-1: Enabled/vasodilator-stimulated phosphoprotein homology 1
EGFP: Enhanced green fluorescent protein
OGD: Oxygen–glucose deprivation
AdVs: Adenoviruses
DIV: Days in vitro
ANOVA: Analysis of variance
